# A Modified Higher-Order Singular Value Decomposition Framework With Adaptive Multilinear Tensor Rank Approximation for Three-Dimensional Magnetic Resonance Rician Noise Removal

**DOI:** 10.3389/fonc.2020.01640

**Published:** 2020-09-11

**Authors:** Li Wang, Di Xiao, Wen S. Hou, Xiao Y. Wu, Lin Chen

**Affiliations:** ^1^Key Laboratory of Biorheological Science and Technology of Ministry of Education, Chongqing University, Chongqing, China; ^2^Australian e-Health Research Centre, Commonwealth Scientific and Industrial Research Organisation (CSIRO), Brisbane, QLD, Australia; ^3^Chongqing Key Laboratory of Artificial Intelligence and Service Robot Control Technology, Chongqing, China; ^4^Collaborative Innovation Center for Brain Science, Chongqing University, Chongqing, China; ^5^Chongqing Medical Electronics Engineering Technology Research Center, Chongqing University, Chongqing, China

**Keywords:** HOSVD, logarithm function, low rank tensor approximation, Rician noise, magnetic resonance images

## Abstract

The magnetic resonance (MR) images are acknowledged to be inevitably corrupted by Rician distributed noise, which adversely affected the image quality for diagnosis purpose. However, the traditional denoising methods may recover the images from corruptions with severe loss of detailed structure and edge information, which would affect the lesion detections and diagnostic decision making. In this study, we challenged improving the Rician noise removal from three-dimensional (3D) MR volumetric data through a *modified higher-order singular value decomposition* (*MHOSVD*) method. The proposed framework of *MHOSVD* involved a parameterized logarithmic nonconvex penalty function for low-rank tensor approximation (LRTA) algorithm optimization to suppress the image noise in MR dataset. Reference cubes were extracted from the noisy image volume, and block matching was performed according to nonlocal similarity for a fourth-order tensor construction. Then the LRTA problem was implemented by tensor factorization approaches, and the ranks of unfolding matrices along different modes of the tensor were estimated utilizing an adaptive nonconvex low-rank method. The denoised MR images were finally restored through aggregating all recovered cubes. We investigated the proposed algorithm *MHOSVD* on both the synthetic and real clinic 3D MR images for Rician noise removal, and relative results demonstrated that the *MHOSVD* can recover images with fine structures and detailed edge preservation with heavy noise even as high as 15% of the maximum intensity. The experimental results were also compared along with several classical denoising methods; the *MHOSVD* exhibited a sufficient improvement in noise-removal performance at various noise conditions in terms of different measurement indices such as peak signal-to-noise ratio and structural similarity index metrics. Based upon the comparison, the proposed *MHOSVD* has proved a relative state-of-the-art performance with excellent detailed structure reservation.

## Introduction

Magnetic resonance imaging (MRI), as a widely accepted noninvasive imaging modality, was acknowledged as a useful diagnostic tool with high resolution and excellent contrast sensitivity to anatomical properties (1). However, despite the significant improvements in instrument technique during recent years, the detection or assessment of specific diagnostic information through referring physicians or computer-aided analysis still frequently suffered from serious random noise. As is known, the assessment of medical image quality is usually described by various physical measures including the contrast between different tissues, the detailed representation relative to imaging spatial resolution, and the image noise characteristics. The introduction of noise into MR images can usually cause imaging blur or fine structure coverage/distortion that may severely degrade the image quality and affect the diagnostic accuracy. As reported, a high level of noise may directly affect the signals resulting in anatomical inconsistencies that especially correspond to cardiac and brain images or may bias the orientations of tensors in functional MRI. Meanwhile, computer-aided diagnosis (CAD) approaches recently have been developed as assistive tools to help radiologists to detect and clarify abnormalities. Typical CAD systems based on medical images for lesion separation and recognition generally employed techniques including segmentation, feature extraction, and classification. However, one key problem that challenges the accuracy of CAD tasks was sensitivity to artifacts such as noise. Hence, to improve the performance of CAD approaches, image preprocessing was necessary wherein proper denoising algorithms were essential for the reliability and robustness of computer-aided detection/diagnosis. An ideal denoising method should restore the images by removing the stochastic corruptions of noise with preservation of the shapes and detailed structures of the tissue against abnormities as well. Previous studies have considered the complex raw data of MRI images be corrupted by white Gaussian noise with zero mean and same variance in both the real and imaginary parts; thus, the noise characters were transformed into Rician distribution in magnitude images (2, 3). Therefore, an effective denoising algorithm specific for Rician noise based upon three-dimensional (3D) MR image datasets can play a fundamental role to improve the diagnostic accuracy for both the radiologists and the CAD tools.

To date, several techniques have generally been implemented in commercial MRI devices to improve the signal-to-noise ratio (SNR) for image quality control (4). Along with the hardware evolutions such as magnetic field intensity that dramatically increased, efficient imaging processing algorithms were also utilized to recover the undesirable random variations that obscured the diagnostic information. One applicable way of denoising was to take the average from multiple repeated acquisitions, with the price of screening time extension, which was difficult for patients to keep static. The more popular way was to involve the reduction of noise power level during post-imaging processing. As MRI intrinsically posed a paradox between SNR and resolution, the ideal noise removal procedure should be able to perform SNR improvements without harming the fine structures and detailed features in images. Numerous methods for MR image denoising have been developed based upon different characters of noise. Filter-based approaches constructed the noise-reduction schemes with linear or nonlinear filters, such as anisotropic diffusion and total variation (TV) techniques, to improve image quality with edge preservation considerations (5–8). A few other studies utilized the statistics/estimation of noise properties to conduct denoising; examples included maximum likelihood (9, 10), linear minimum mean square error (11, 12), and phase error (13), which were generally used as estimators for Rician noise. Recently, the transform approaches were demonstrated to be powerful by exploiting the different representations of noise against signal either spatially or spectrally in transform domain (14–16). For example, wavelet transforms were used to decompose the signal and noise into multiresolution subspaces, which facilitated the Rician noise removal while preserving edge and fine details (17). Besides, curvelet transform and contourlet transforms were also used for denoising MR images (18, 19). Moreover, deep learning methods were also involved in the Rician noise removal in MR images. For example, Jiang et al. (20) developed a multichannel conventional neural network (CNN) method for noise removal in synthetic and clinical MR volumetric image. Manjón et al. proposed a two-step 3D MR image denoising algorithm by combining CNN with rotation-invariant nonlocal means (NLM) filtering. The relative results were encouraging, which improve the visual quality significantly; however, the deep leaning frameworks still need to meet the high burdens of huge training dataset and time-consuming computation (21).

More recent studies have intended to combine the strengths of multiple approaches (e.g., filter and transform approaches) to better regulate denoising in MRI (22, 23). The well-known NLM filter that Buades et al. (24) proposed was one classical method that considered exploiting nonlocal patch similarity for noise removal (24). The redundancy patterns within images ensured useful information to be restored by NLM with outstanding edge sharpness maintained. Kervrann et al. (25) soon extended the NLM algorithm into variations combined with transform approaches with the purpose of taking advantage of both similarity and sparsity. One famous NLM variation was block matching 3D (BM3D), which constructed its scheme by grouping image fragments according to similarity, filtering with the sparse representation in transform domain, and transforming back by aggregation procedure (26). Maggioni et al. (27) then modified the BM3D method using NLM and cosine/wavelet transform into BM4D implementation, and they demonstrated state-of-the-art in volumetric MRI data recovery. Meanwhile, the development in tensor factorization, such as the higher-order singular value decomposition (SVD) (HOSVD) (28), enabled better sparse representations using learned basis rather than the fixed orthogonal basis that cosine/wavelet transforms generally used in BM3D/BM4D. Rajwade et al. (29) derived the HOSVD denoising algorithm, which manipulated the NLM filtering with HOSVD transform instead. As the learning basis was adaptable to image contents, redundant procedures of patches rearrangements were saved, and further performance improvements were promising. In Zhang et al. (30) have extended the HOSVD algorithm for MRI, and excellent noise-reduction effectiveness was obtained. However, the HOSVD-based denoising method generally computed the transform coefficients using hard threshold function with nonconvex and nonlinear properties, which was considered to constrain the denoising performance somehow. Further, the hard threshold function would also cause additional shock and pseudo-Gibbs effect, which may limit the retention of image structure.

The present study aimed to improve the performance of HOSVD application for denoising 3D MR volumetric data. To address the drawback mentioned above, we modified the HOSVD algorithm [named as modified HOSVD (*MHOSVD*)] with an adaptive multilinear tensor rank approximation method utilizing nonlocal similarity and nonconvex logarithmic regularization. Image cubes from volumetric data were stacked based on similarity to construct a fourth-order tensor. Then tensor factorization was performed. A low-rank tensor was derived to approximate the sparse representations by exploiting the image structural redundancy. We estimated the ranks of unfolding matrices along different modes of the tensor using an adaptive nonconvex low-rank method. Experiments were performed based on various MR images, obtained from either computer synthesis or clinical screening, to investigate denoising improvements of proposed framework against several advanced algorithms with the following novelties:

1. Considered the nonlocal similarity in 3D MR datasets and formulated the grouped similar cubes into a low-rank tensor approximation (LRTA) problem.
2. Applied a nonconvex low-rank function to adaptively estimate the rank of unfolding matrices along different modes.
3. Used the proximal operator to obtain the global closed-form solution for the constructed low-rank approximation problem.

We structured the rest of this manuscript as follows: *Background* reviews necessary backgrounds about the tensor. *Proposed Model* describes our algorithm proposed for Rician noise removal based upon adaptive HOSVD framework. We validated the proposed schemes to both synthetic and clinical 3D MR images, and the effectiveness of noise reduction was evaluated and compared with that of existing denoising algorithms in *Experiments and Results*. Last but not the least, we draw our conclusion in *Conclusions*.

## Background

A tensor can be considered as a generalization of vector and matrix for the representation of multidimensional quantities. Throughout the article, we represented the scalars as lowercase letters (e.g., x), vectors as bold lowercase letters (e.g., **x**), matrices as bold uppercase letters (e.g., **X**), and tensors as bold calligraphic letters (e.g., X). For example, the symbol $X\in R^{J_{1}\times J_{2}\times \ldots \times J_{N}}$ signified an arbitrary *N*th-order tensor over the multidimensional space of size *J*_1_ × *J*_2_ × … × *J*_*N*_. We also used the subscript lowercase letters to present the element tensor; e.g., an arbitrary element of the tensor X can be denoted as $X_{j_{1}j_{2}\ldots j_{N}}$ using (*j*_1_*j*_2_…*j*_*N*_).

**Definition 1** (Tensor fiber). A tensor generally consists of fibers that can be thought as the high-dimensional generalization of matrix rows and columns. The mode-*n* fiber of tensor X is defined by fixing the index in all dimensions but the *n*-th dimension.

**Definition 2** (Tensor unfolding). Tensor unfolding, also called tensor matricization, is to rearrange the elements of the tensor into the matrix format under predefined order. The mode-*n* matricization of an *N*th-order tensor $X\in R^{J_{1}\times J_{2}\times \ldots \times J_{N}}$ would be represented as ${\text{X}}_{\left(n\right)}\in R^{J_{n}\times \left(J_{1}\times \ldots \times J_{n-1}\times J_{n+1}\times \ldots \times J_{N}\right)}$, which ordered the mode-*n* fibers of tensor X into its columns.

**Definition 3** (*n*-mode matrix product). The product of a tensor $X\in R^{J_{1}\times J_{2}\times \ldots \times J_{N}}$ and a matrix $\text{V}\in R^{K\times J_{n}}$ along the *n*-th dimension is a tensor of size *J*_1_ × … × *J*_*n*−1_ × *K* × *J*_*n*+1_ × … × *J*_*N*_, denoted as $X\times_{n}\text{V}$, with its element expressed by following equation:

(1) $$\begin{array}{r} {\left(X\times_{n}\text{V}\right)}_{j_{1}\ldots j_{n-1}kj_{n+1}\ldots j_{N}}=\overset{J_{n}}{\underset{j_{n}=1}{\sum }}x_{j_{1}j_{2}\ldots j_{N}}v_{kj_{n}}. \end{array}$$

**Definition 4** (Multilinear tensor rank). The multilinear tensor rank of $X\in R^{J_{1}\times J_{2}\times \ldots \times J_{N}}$ is defined as the rank of the mode-*n* unfolding matrix **X**_(*n*)_, denoted as $rank_{n}\left(X\right)$.

## Proposed Model

### Low-Rank Matrix Approximation

Low-rank matrix approximation (LRMA) aimed to solve the minimization problem through optimizing the cost function between the given data and an approximation with reduced rank. The method was helpful in various image processing such as 2D image denoising (31, 32). The LRMA usually applied for image noise removal following steps, as follows: searching and grouping nonlocal similar patches, performing collaborative filtering, and aggregating the recovered patches to restore the denoised images. Mathematically, the LRMA method can be modeled as follows:

(2) $${}_{X\;}^{\min}\;\frac{1}{2}\|Y-X{\|}_{F}^{2}+\lambda rank(X),$$

where rank(**·**) represented the rank of matrix, referring to the number of nonzero singular values of matrix. λ was the trade-off parameter balancing the contribution between fidelity and regularization term. However, due to the high nonconvex and nonlinear properties, problem (Eq. 2) was practically intractable. Therefore, convex relaxation nuclear norm was used instead, and problem (Eq. 2) can be transformed into the following LRMA problem:

(3) $${}_{X\;}^{\min}\;\frac{1}{2}{\left\|Y-X\right\|}_{F}^{2}+\lambda {\left\|X\right\|}_{*}\;,$$

where ${\left||\text{X}|\right|}_{*}=\underset{i}{\sum }\delta_{i}\left(\text{X}\right)$ denoted the nuclear norm that was defined as the sum of a singular value from matrix **X**. Cai et al. (33) have proved that the solution of the LRMA problem (Eq. 3) can be conveniently obtained by singular value thresholding; i.e., $\overset{\hat{\;}}{X}=US_{\lambda }\left(\Sigma \right)V^{\text{T}}$, where **Y** = **UΣV**^*T*^ was the SVD of the noisy image **Y**, and *S*_λ_(**Σ**) = max(**Σ**−λ, 0).

### Low-Rank Tensor Approximation

LRTA can extend the LRMA algorithm for multidimensional image processing. In this study, we employed the LRTA for 3D MR image denoising. First, reference cube sized of *p* × *p* × *p* was extracted from the MR noisy image. Block matching was performed with a local search window of size *k* × *k* × *k*, and *m* similar cubes (including the reference cube) were found and stacked to a fourth-order tensor $Y\in R^{p\times p\times p\times m}$. Then the problem estimating the noise-free version X according to noisy image Y can be regarded as an LRTA mathematically modeled as

(4) $$\begin{array}{r} X^{*}{=}_{X_{}}^{argmin}\;\frac{1}{2}{||Y-X|{|}_{F}^{2}\;s.t.\;rank}_{i}\left(X\right)\leq r_{i}\;\left(i=1,2,3,4\right),\; \end{array}$$

where $rank_{i}\left(X\right)$ denoted the rank of the mode-*i* unfolding matrix of the fourth-order tensor X. Based upon the HOSVD (28), tensor X can be decomposed into the following:

(5) $$\begin{array}{r} X=S\times_{1}{\text{U}}^{\left(1\right)}\times_{2}{\text{U}}^{\left(2\right)}\times_{3}{\text{U}}^{\left(3\right)}\times_{4}{\text{U}}^{\left(4\right)}, \end{array}$$

where $S\in R^{r_{1}\times r_{2}\times r_{3}\times r_{4}}$ was the core tensor and ${\text{U}}^{\left(i\right)}\in R^{p\times r_{i}}\;\left(i\;=\;1,2,3,4\right)$ was the factorization matrix. The key for solving problem (Eq. 5) was to estimate the multilinear tensor rank parameter *r*_*i*_(*i* = 1, 2, 3, 4). Therefore, the LRTA problem (Eq. 4) can be independently decomposed into four relative minimization problems:

(6) $$\begin{array}{r} X_{\left(n\right)}^{*}{=}_{X_{\left(n\right)\;}}^{argmin}\;\frac{1}{2}|\left|Y_{\left(n\right)}-X_{\left(n\right)}\right|{|}_{F}^{2}+\lambda rank(X_{\left(n\right)})\;\left(n=1,2,3,4\right), \end{array}$$

where *rank*(**X**_(*n*)_) was the number of nonzero singular values of the mode-*n* unfolding matrix **X**_(*n*)_, and ${\left||Y-X|\right|}_{F}^{2}={\left||{\text{Y}}_{\left(n\right)}-{\text{X}}_{\left(n\right)}|\right|}_{F}^{2}$ were the Frobenius norm.

### Adaptive Multilinear Tensor Rank Algorithm

As the solution of problem (Eq. 6) was intractable, the nuclear norm with nonconvex properties have been demonstrated an effective tool for sparsity of singular values compared with the convex rank function. Motivated by Selesnick and Bayram (34), Chen and Selesnick (35), and Parekh and Selesnick (36), we propose a parameterized logarithmic nonconvex penalty function on singular values, formulated as follows:

(7) $$\begin{array}{r} X_{\left(n\right)}^{*}{=}_{X_{\left(n\right)\;}}^{argmin}\;\frac{1}{2}|\left|Y_{\left(n\right)}-X_{\left(n\right)}\right|{|}_{F}^{2}+\lambda \sum_{i=1}^{r_{n}}\frac{1}{a}\log\left(1+a\delta_{i}\left(X_{\left(n\right)}\right)\right), \end{array}$$

where δ_*i*_(**X**_(*n*)_) was the *i*-th singular value of the mode-*n* unfolding matrix **X**_(*n*)_. Although the nonconvex penalty function was employed, the proposed LRTA problem (Eq. 7) was strictly convex when 0 ≤ *a* < 1/λ, which could be proven by *Lemma 1*, as follows.

*Lemma* 1(37): The nonconvex function $\psi \left(x,a\right)=\frac{1}{a}log\left(1+ax\right)$ satisfied Assumption 1 as mentioned in literature (36). The function h: *R*→*R* represented as *h*(*x, a*) = ψ(*x, a*)−|*x*| was continuously differentiable and concave and satisfied −*a* ≤ *h*″(*x, a*) ≤ 0.

Eq. (7), which was considered to be strictly convex when 0 ≤ *a* < 1/λ, was proved, as follows.

Proof: Define the function J:*R*^*m* × *n*^→*R* as

$$\begin{array}{l} \begin{array}{c} J\left(X\right)=\frac{1}{2}\|Y-X{\|}_{F}^{2}+\lambda \overset{r_{n}}{\underset{i=1}{\sum }}\left(h\left(\delta_{i}\left(X\right),a\right)+|\delta_{i}\left(X\right)|\right)\\ \;=\frac{1}{2}tr\left(Y^{T}Y\right)-tr\left(XY^{T}\right)+\frac{1}{2}tr\left(X^{T}X\right)\\ \;+\lambda \overset{r_{n}}{\underset{i=1}{\sum }}\left(h\left(\delta_{i}\left(X\right),a\right)+|\delta_{i}\left(X\right)|\right)\\ \;=\frac{1}{2}tr\left(Y^{T}Y\right)-tr\left(XY^{T}\right)+\frac{1}{2}tr\left(X^{T}X\right)\\ \;+\lambda \overset{r_{n}}{\underset{i=1}{\sum }}h\left(\delta_{i}\left(X\right),a\right)+\lambda \|X{\|}_{*}\\ \;=J_{1}\left(X\right)+\frac{1}{2}tr\left(Y^{T}Y\right)-tr\left(XY^{T}\right)+\lambda \|X{\|}_{*}, \end{array} \end{array}$$

where $J_{1}\left(X\right)=\frac{1}{2}tr\left(X^{T}X\right)+\lambda \sum_{i=1}^{r_{n}}h\left(\delta_{i}\left(X\right),a\right)$. Since both the linear function *tr*(*XY*^*T*^) and trace norm ||*X*||_*_ were convex function and the function *tr*(*Y*^*T*^*Y*) was independent on X, the *J*(*X*) was strictly convex when the function *J*_1_(*X*) was strictly convex. According to Parekh and Selesnick (36), we can conclude that the function *J*_1_(*X*) was strictly convex when 0 ≤ *a* < 1/λ.

According to Selesnick and Bayram (34), the proximal operator of the proposed penalty function (Eq. 7) was defined as:

(8) $$\begin{array}{r} \theta \left(y;\lambda ,a\right){=}_{x\in R\;}^{argmin}\;\frac{1}{2}{\left(y-x\right)}^{2}+\lambda \frac{1}{a}\log\left(1+ax\right), \end{array}$$

and the equivalent threshold function of the proximal operator (8) can be defined as (34, 38)

(9) $$\theta \left(y;\lambda ,a\right)=\{\begin{array}{c} \frac{\left|y\right|}{2}-\frac{1}{2a}+\sqrt{{\left(\frac{\left|y\right|}{2}+\frac{1}{2a}\right)}^{2}-\frac{\lambda }{a}}\;\left|y\right|\geq \lambda .\\ 0\;|y|<\lambda \end{array}\;$$

When the proximal operator θ(•) was applied to matrix **X**, the element-wise would need to be treated. Then the proposed LRTA problem (Eq. 7) can globally solved with optimal solution:

(10) $$\begin{array}{r} {\text{X}}_{\left(n\right)}^{*}={\text{U}}^{\left(n\right)}\theta \left(\Sigma^{\left(n\right)};\lambda ,a\right){\left({\text{V}}^{\left(n\right)}\right)}^{T}, \end{array}$$

where ${\text{Y}}_{\left(n\right)}={\text{U}}^{\left(n\right)}\Sigma^{\left(n\right)}{\left({\text{V}}^{\left(n\right)}\right)}^{T}$. Therefore, the multilinear tensor rank parameter *r*_*n*_ can be adaptively estimated as the number of the nonzero elements in θ(**Σ**^(*n*)^; λ, *a*), and the estimated factorization matrix ${\overset{\hat{\;}}{\text{U}}}^{\left(\text{n}\right)}$was selected as the first *r*_*n*_ columns of the **U**^(*n*)^. Thus, the estimated core tensor can be formulated as:

(11) $$\begin{array}{r} \overset{\hat{\;}}{S}=Y\times 1{\left({\overset{\hat{\;}}{U}}^{\left(1\right)}\right)}^{\text{T}}\times 2{\left({\overset{\hat{\;}}{U}}^{\left(2\right)}\right)}^{\text{T}}\times 3{\left({\overset{\hat{\;}}{U}}^{\left(3\right)}\right)}^{\text{T}}\times 4{\left({\overset{\hat{\;}}{U}}^{\left(4\right)}\right)}^{\text{T}}, \end{array}$$

and the restored tensor $\overset{\hat{\;}}{X}$ can be obtained by:

(12) $$\begin{array}{r} \overset{\hat{\;}}{X}=\overset{\hat{\;}}{S}\times 1{\left({\overset{\hat{\;}}{U}}^{\left(1\right)}\right)}^{\text{T}}\times 2{\left({\overset{\hat{\;}}{U}}^{\left(2\right)}\right)}^{\text{T}}\times 3{\left({\overset{\hat{\;}}{U}}^{\left(3\right)}\right)}^{\text{T}}\times 4\left({\overset{\hat{\;}}{U}}^{\left(4\right)}\right)\text{T}. \end{array}$$

### Variance-Stabilizing Transform

MR images were mainly contaminated by Rician-distributed noise (3). For Rician noise removal in 3D MR image, we implemented the forward and inverse variance-stabilizing transform (VST) (38) into our proposed denoising framework. The forward VST was used to convert the 3D MR images with signal-dependent Rician-distribution noise into the new volumetric data with homoscedastic Gaussian-distributed noise. Once the proposed denoising algorithm was applied, the inverse VST (*VST*^−1^) counteracted the recovered data to obtain the denoised images. Therefore, the whole scheme of the proposed denoising algorithm in 3D MR images can be formulated as:

(13) $$\begin{array}{r} \overset{\hat{\;}}{X}=\text{VS}{\text{T}}^{-1}\left(\text{Denoising}\left(\text{VST}\left(Y\text{,}\sigma_{\text{n}}\right)\text{,}\sigma_{\text{VST}}\right)\text{,}\sigma_{\text{n}}\right), \end{array}$$

where Y is the 3D MR images contaminated by Rician-distributed noise, σ_*n*_ represents the Rician noise level, and σ_*VST*_ is the standard deviation of noise after VST. We describe the proposed denoising method in Algorithm 1.

**Algorithm 1:** 3D MR image denoising

**Input:** 3D MR noise images $Y_{\text{noise}}$
**Output:** Denoised MRI image X
**Initialization:** X=Y
for t = 1,2,…T do
Iterative regularization: $Y^{\text{(t)}}=X^{\text{(t-1)}}+\beta \left(Y-X^{\text{(t-1)}}\right)$
Update the noise deviation σ_*VST*_ according to $\sigma_{\text{VST}}=\gamma \sqrt{{\sigma_{\text{VST}}}^{2}-||Y-Y^{\left(t\right)}|{|}_{\text{F}}^{2}}$
Search for similar patches and group them to form fourth-order tensor Y for each reference patch;
Decompose each tensor by Eq. (5) and compute θ(**Σ**^(*n*)^; λ, *a*) by Eq. (9);
Update the factorization matrix **U**^(*i*)^ (*i* = 1, 2, 3, 4);
Compute the core tensor S by Eq. (11);
Compute the denoised tensor $\overset{\hat{\;}}{X}$ by Eq. (12);
Aggregating all $\overset{\hat{\;}}{X}$ to obtain denoised image;
End for

## Experiments and Results

In this section, we validated the performance of the proposed algorithm (refer as *MHOSVD*) on both the synthetic and clinical 3D MR images. The noise-free MR images for noise synthesis, including T1-weighted (T1w) data, T2-weighted (T2w) data, and proton density-weighted (PDw) data, were downloaded from BrainWeb database (39, 40). The raw data were formatted in a size of 181 × 217 × 181 with 1 mm^3^ × 1 mm^3^ × 1 mm^3^ voxel resolution. The noise data were simulated by adding different levels of spatially invariant Rician noise (from 1% to 15% of the maximum intensity with an increase of 2%).

The peak SNR (PSNR) and structural similarity index measurement (SSIM) were calculated to quantitatively evaluate the denoising performance. PSNR was a metric measuring the ratio of the maximum possible signal power to the noise power, which is defined as:

(14) $$\begin{array}{r} PSNR=10\log_{10}\frac{MAX^{2}}{MSE}, \end{array}$$

where *MSE* is the mean squared error (*MSE*) between the denoised image and ground truth and *MAX* represents the maximum image intensity. According to the definition, a higher PSNR value would indicate lower noise content that related to a better image quality.

Meanwhile, the metric of SSIM was defined according to human eye perception:

(15) $$\begin{array}{r} SSIM=\frac{\left(2\mu_{x}\mu_{x^{*}}+c_{1}\right)\left(2\sigma_{x}\sigma_{x^{*}}+c_{2}\right)}{\left(\mu_{x}^{2}+\mu_{x^{*}}^{2}+c_{1}\right)\left(\sigma_{x}^{2}+\sigma_{x^{*}}^{2}+c_{2}\right)}\;, \end{array}$$

where μ_*x*_ and σ_*x*_ denote the mean and standard deviation of the ground truth image, respectively, while the $\mu_{x^{*}}$ and $\sigma_{x^{*}}$ denote the mean and standard deviation of the denoised image, respectively. *c*_1_ and *c*_2_ are constants. Therefore, the range of SSIM was from −1 to 1. An SSIM value close to 1 referred to a perfect similarity comparing the denoised image with the ground truth, which resulted in a higher performance of image restoration.

### Parameter Sets

The parameters that may determine the denoising performance of the proposed algorithm (*MHOSVD*) included the following: the size of cube *p*, the number of similar cubes in a group *m*, the size of searching volume *L*, and noise-feedback parameters β and γ. A large value of *p* and *m* tended to benefit the removal of image data corruption at high-level noise. However, a large value of parameter *m* that denoted the number of similar cubes would also result in intragroup dissimilar cubes as a trade-off. Efforts were paid to balance the advantages and disadvantages; the setting of parameters *p* and *m* at different noise levels was chosen and shown in Table 1. Similarly, the increase in searching volume parameter *L* can help better in cube grouping according to image similarity for higher PSNR and also with a cost of the computational burden. The size of the search window (*L*) was set as 13 according to previous studies (27). The noise-feedback parameters β and γ, which were critical to the feedback of the residual image and residual noise, were set to 0.65 and 0.2 on the basis of previous research (30), respectively. In addition, the parameter *a* from parameterized logarithmic nonconvex penalty function (Eq. 7) and threshold τ from the proximal operator (9) were set as 0.5/τ and 2 × *log*(*p*^3^*m*), respectively.

**Table 1:** The setting of the cube size *p* and numbers of similar cubes *m* at varying noise levels.

**Noise level**	**1%**	**3%**	**5%**	**7%**	**9%**	**11%**	**13%**	**15%**
*p*	3	3	4	4	5	5	5	5
*m*	50	75	75	75	90	90	90	90

### Denoising on Synthetic Three-Dimensional Magnetic Resonance Images

**Figure 1:**
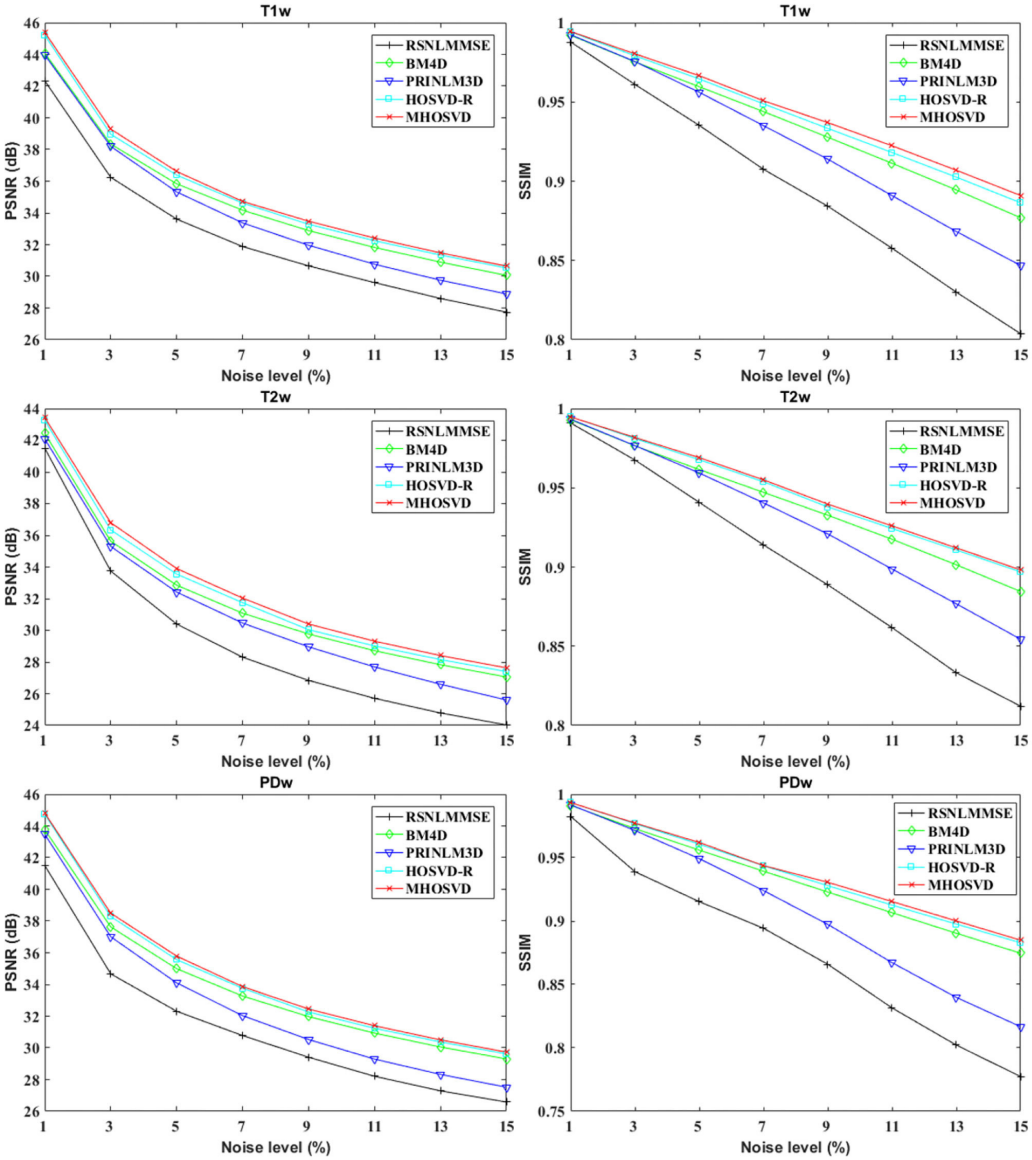
Peak signal-to-noise ratio (PSNR) and structural similarity index measurement (SSIM) comparisons across four denoising methods (RSNLMMSE, BM4D, PRI-NLM3D, HOSVD-R, and *MHOSVD*) for Rician noise removal in three-dimensional magnetic resonance (3D MR) images synthesized with corruptions under different noise levels.

The performance of proposed algorithm (*MHOSVD*) was first tested on synthetic brain 3D MR images, against several existing classical algorithms including RSNLMMSE (11), BM4D (27), PRI-NLM3D (22), and HOSVD-R (30). The corresponding PSNR performance and SSIM performance on T1w, T2w, and PDw images were compared across algorithms. As illustrated by Figure 1, the proposed *MHOSVD* exhibited encouraging improvements over the other three methods, which had been previously verified to be state-of-the-art. Further, we also tabulated the PSNR (Table 2) and SSIM (Table 3) obtained by these four denoising algorithms (RSNLMMSE, BM4D, PRI-NLM3D, HOSVD-R, and *MHOSVD*) on MR images (T1w, T2w, and PDw) adding Rician noise under different noise levels (1, 3, 5, 7, 9, 11, 13, and 15%, respectively). The proposed *MHOSVD* outperformed RSNLMMSE (ranged from 1.9337 to 3.8249 dB), PRI-NLM3D (1.091 to 2.2149 dB), BM4D (0.4305 to 1.3077 dB), and HOSVD-R (0.0737 to 0.46 dB) with regard to PSNR. The same superiority was also observed according to the metric SSIM, indicating that the proposed *MHOSVD* can better remove Rician noise with structure preservation against corruptions across a board range of noise level.

**Table 2:** Peak signal-to-noise ratio (PSNR) comparisons across RSNLMMSE, BM4D, PRI-NLM3D, HOSVD-R, and MHOSVD for Rician noise removal in synthetic three-dimensional magnetic resonance (3D MR) images.

**Noise level**	**1%**	**3%**	**5%**	**7%**	**9%**	**11%**	**13%**	**15%**
**T1w**								
RSNLMMSE	42.3349	36.2450	33.6064	31.8756	30.6574	29.6012	28.6028	27.7378
BM4D	44.0842	38.3433	35.8339	34.1703	32.8852	31.8209	30.8960	30.0623
PRI-NLM3D	43.9625	38.1993	35.3249	33.3687	31.9575	30.7627	29.7537	28.8873
HOSVD-R	45.2072	38.9638	36.3855	34.6173	33.2822	32.2407	31.3391	30.5257
MHOSVD	**45.3919**	**39.2903**	**36.6157**	**34.7226**	**33.4781**	**32.4122**	**31.4775**	**30.6433**
**T2w**								
RSNLMMSE	41.5029	33.7452	30.3948	28.3027	26.8397	25.7065	24.7832	24.0270
BM4D	42.4365	35.6338	32.8497	31.0817	29.7697	28.7149	27.8282	27.0561
PRI-NLM3D	42.0810	35.3006	32.4165	30.4594	28.9082	27.6894	26.5963	25.6028
HOSVD-R	43.2601	36.3387	33.5516	31.7446	30.0404	29.0155	28.1549	27.3969
MHOSVD	**43.4366**	**36.7987**	**33.9049**	**32.0353**	**30.3983**	**29.3149**	**28.4077**	**27.6288**
**PDw**								
RSNLMMSE	41.5061	34.6722	32.3071	30.7760	29.4088	28.2082	27.2879	26.5824
BM4D	43.7633	37.6312	34.9947	33.2691	31.9738	30.9310	30.0547	29.3031
PRI-NLM3D	43.4725	37.0113	34.0964	32.0319	30.5257	29.2910	28.3261	27.5187
HOSVD-R	44.7386	38.2867	35.5647	33.7573	32.2607	31.2341	30.3690	29.6179
MHOSVD	**44.8123**	**38.4971**	**35.7897**	**33.8636**	**32.4501**	**31.3949**	**30.5037**	**29.7336**

*The bold values in represented the maximum values across the methods*.

**Table 3:** Structural similarity index measurement (SSIM) comparisons across BM4D, PRI-NLM3D, HOSVD-R, and MHOSVD for Rician noise removal in synthetic three-dimensional magnetic resonance (3D MR) images.

**Noise level**	**1%**	**3%**	**5%**	**7%**	**9%**	**11%**	**13%**	**15%**
**T1w**								
RSNLMMSE	0.9878	0.9610	0.9352	0.9075	0.8842	0.8577	0.8300	0.8038
BM4D	0.9921	0.9753	0.9596	0.9439	0.9278	0.9113	0.8945	0.8770
PRI-NLM3D	0.9924	0.9755	0.9561	0.9350	0.9139	0.8909	0.8683	0.8469
HOSVD-R	0.9942	0.9791	0.9645	0.9488	0.9333	0.9181	0.9026	0.8864
MHOSVD	**0.9944**	**0.9804**	**0.9666**	**0.9508**	**0.9370**	**0.9223**	**0.9069**	**0.8910**
**T2w**								
RSNLMMSE	0.9909	0.9675	0.9408	0.9139	0.8887	0.8617	0.8332	0.8121
BM4D	0.9930	0.9765	0.9615	0.9471	0.9327	0.9175	0.9014	0.8845
PRI-NLM3D	0.9933	0.9766	0.9563	0.9405	0.9209	0.8987	0.8768	0.8544
HOSVD-R	**0.9948**	0.9811	0.9677	0.9538	0.9379	0.9243	0.9107	0.8969
MHOSVD	0.9947	**0.9818**	**0.9690**	**0.9550**	**0.9397**	**0.9260**	**0.9120**	**0.8981**
**PDw**								
RSNLMMSE	0.9825	0.9389	0.9153	0.8943	0.8659	0.8315	0.8022	0.7774
BM4D	0.9912	0.9729	0.9559	0.9393	0.9229	0.9066	0.8904	0.8747
PRI-NLM3D	0.9916	0.9714	0.9492	0.9240	0.8974	0.8672	0.8398	0.8167
HOSVD-R	**0.9934**	0.9768	0.9606	0.9436	0.9279	0.9126	0.8976	0.8829
MHOSVD	**0.9934**	**0.9772**	**0.9620**	**0.9437**	**0.9306**	**0.9154**	**0.9001**	**0.8849**

*The bold values in represented the maximum values across the methods*.

**Figure 2:**
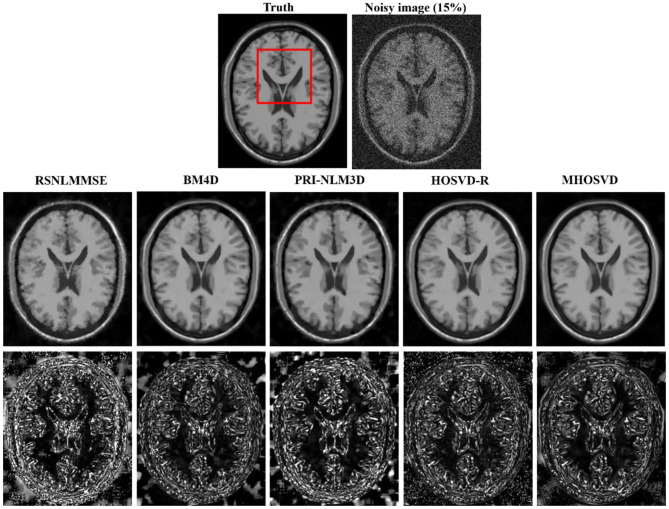
Denoising T1-weighted (T1w) images by different methods (RSNLMMSE, BM4D, PRI-NLM3D, HOSVD-R, and *MHOSVD*) under 15% Rician noise. First line: T2w image without noise and with 15% Rician noise. Second line: the denoised images with four different methods. Third line: the corresponding residual images (the absolute difference between the denoised and noise-free images).

**Figure 3:**
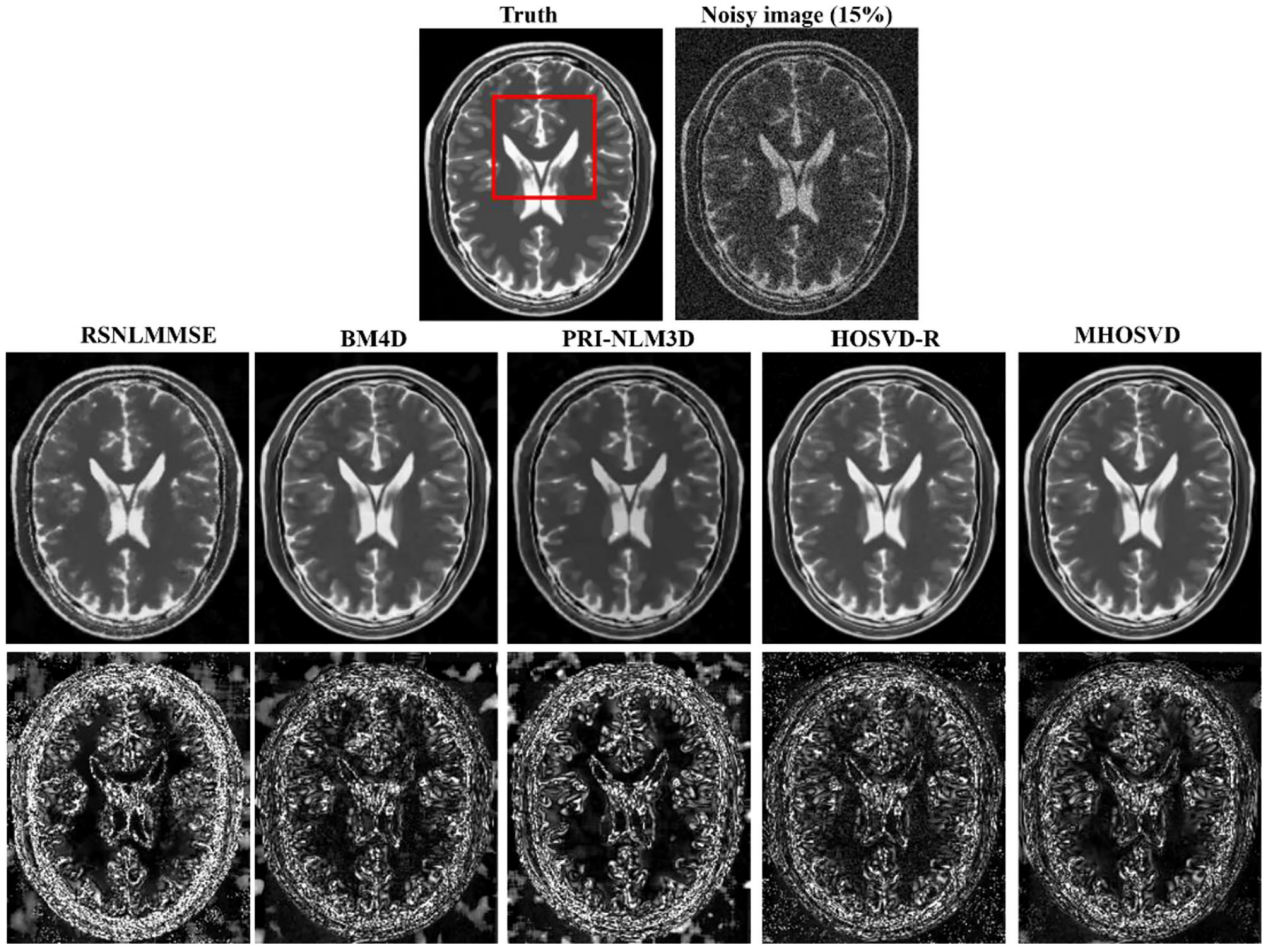
Denoising T2-weighted (T2w) images by different methods under 15% Rician noise. First line: T2w image without noise and with 15% Rician noise. Second line: the denoised images with four different methods. Third line: the corresponding error images.

**Figure 4:**
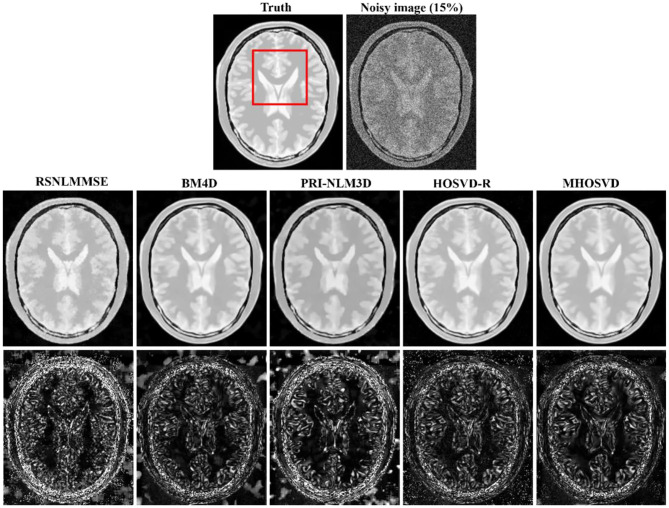
Denoising proton density-weighted (PDw) images by different methods under 15% Rician noise. First line: T2w image without noise and with 15% Rician noise. Second line: the denoised images with four different methods. Third line: the corresponding error images.

**Figure 5:**
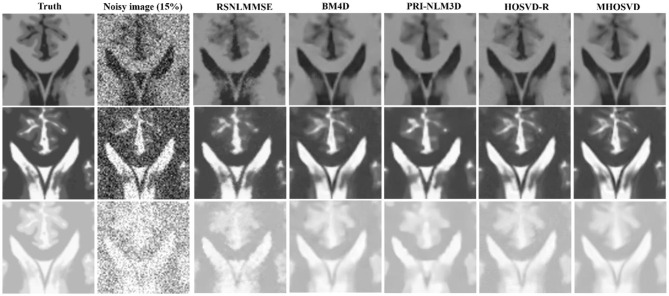
Performance comparison with enlarged region of interest (ROI) visualization using four different methods under 15% Rician noise. ROIs selected from above (red rectangles in Figures 2–4). First line: T1-weighted (T1w) images. Second line: T2-weighted (T2w) images. Third line: proton density-weighted (PDw) images.

Examples of denoising results of all four algorithms on synthesized MR images (T1w, T2w, and PDw) corrupted by the Rician noise at level of 15% are visually shown in Figures 2–4. Through visible observation, the RSNLMMSE and the PRI-NLM3D exhibited a relatively degraded performance as undesirable oversmoothing and higher detailed structure loss were found to be accompanied with noise reduction, than did the other three methods (BM4D, HOSVD-R, and *MHOSVD*). Though all four algorithms showed intensity oscillations in homogenous regions according to the results of residual images (Figures 2–4) and enlarged regions of interest (ROIs) (Figure 5), the proposed *MHOSVD* decreased the unpleasant fluctuation in performance somehow. Nevertheless, the figures illustrate that our algorithm *MHOSVD* retained fine textures pretty well in 3D volumetric images.

**Figure 6:**
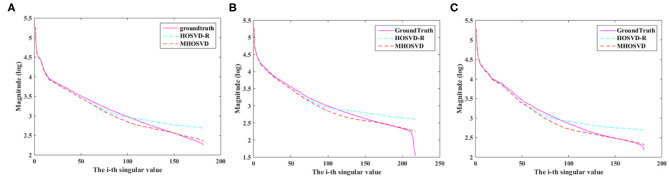
The singular value decomposition (SVD) comparison of different mode unfolding matrices about ground truth and denoised image, destroyed by 15% Rician noise, through the HOSVD-R and the proposed MHOSVD method. **(A)** Mode 1 matrix; **(B)** mode 2 matrix; **(C)** mode 3 matrix.

Compared with hard threshold function, the proposed logarithmic penalty function was closer to the rank function. The singular values on unfolding matrices along different modes also were compared across ground truth and restored images (noisy level set as 15%) via HOSVD-R and MHOSVD method, respectively. As shown in Figure 6, the singular values extracted from recovered images using our proposed method were obviously closer to those of the noise-free images than HOSVD-R, especially in the small singular value domain.

**Figure 7:**
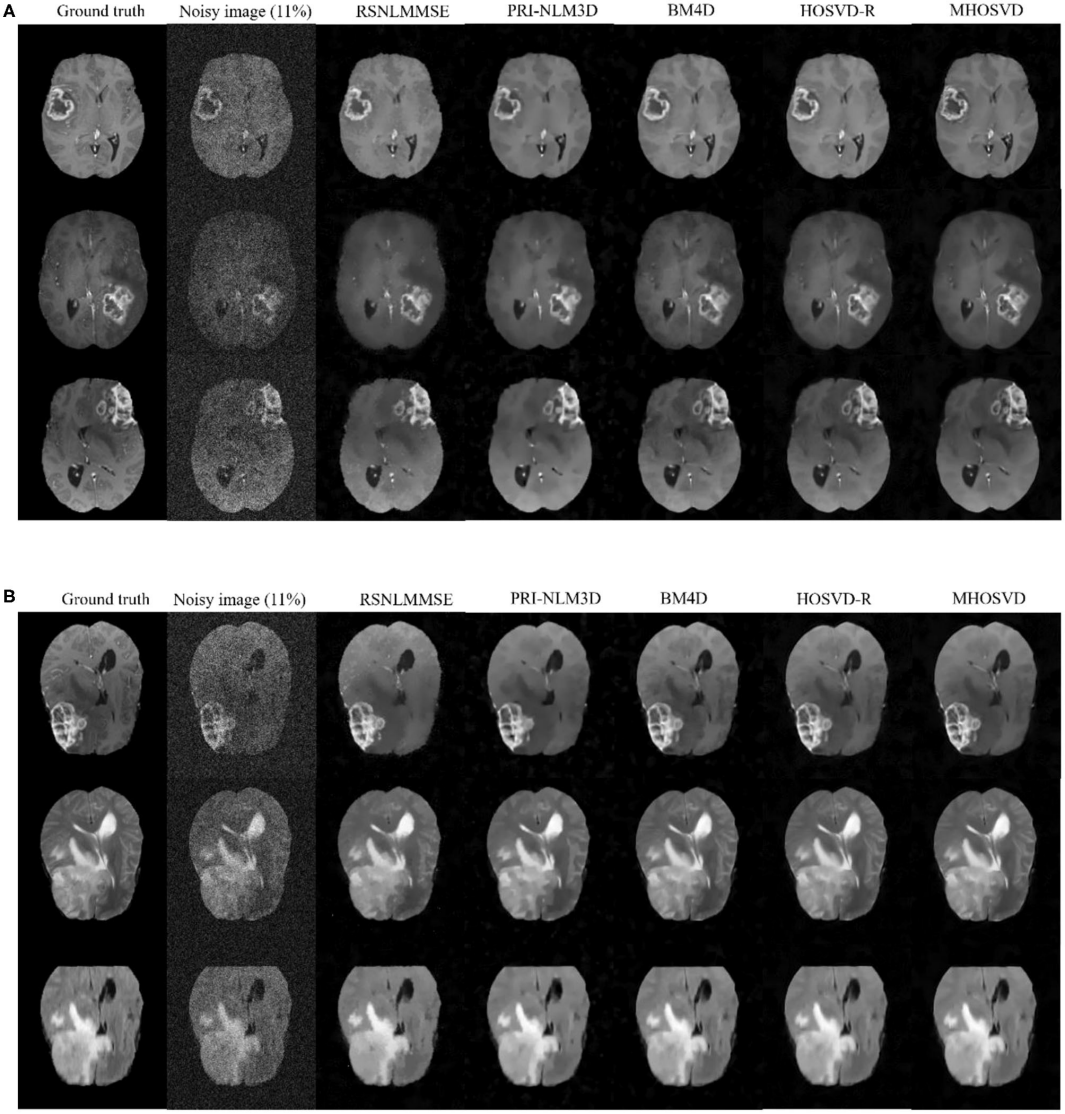
Denoising performance based upon synthesized brain images with abnormities acquired via different MRI modes. The ground truth images (1st column) were downloaded from the open source of BraTS, which included clinical datasets after several image preprocessing approaches. A level of 11% Rician noise was added into the ground truth to synthesize the noised images (second column). The proposed algorithm (MHOSVD) was applied to remove the corruptions against four classical denoising methods (RSNLMMSE, PRI-NLM3D, BM4D, and HOSVD-R), and the results were compared. **(A)** Denoising performances based on T1-weighted contrast-enhanced images acquired from three different patients, and each row corresponds to one patient. **(B)** Denoising performances based on MR datasets acquired by different imaging modes [from top to bottom: T1-weighted contrast-enhanced, T2, and fluid-attenuated inversion recovery (FLAIR)] of the same patient.

In addition, we further applied the proposed algorithm to verify its reliability and effectiveness on the basis of MR images with various abnormities. The ground truth was obtained from the Multimodal Brain Tumor Image Segmentation Challenge (BraTS) 2013, which provided MRI datasets that have already been optimized by several image preprocessing approaches for further application simulations such as CAD segmentations (41). Figure 7 shows some examples randomly selected from the BraTS, as Figure 7A focuses on T1w contrast-enhanced modes from three different patients, while Figure 7B involves a specific case imaged via different modes including T1w contrast enhanced, T2w, and fluid-attenuated inversion recovery (FLAIR). In this study, Rician noise with a level of 11% was introduced into the BraTS data to synthesize the noisy images. As illustrated by the second column of Figure 7, the boundary of lesions can be severely blurred with the impact of noise, and some small structures were even buried by the stochastic variances that noise introduced. The proposed algorithm (*MHOSVD*) was applied to remove the image corruption and restore the image qualities, and the relative results were compared with those of several classical denoising method including RSNLMMSE, PRI-NLM3D, BM4D, and HOSVD-R. Figure 7 illustrates that all algorithms can repair images with different noise removal abilities. Comparing columns 3–7 with column 2, it can be visibly observed that the blurry images were degraded after the denoising procedure (Figure 7), the boundary of pathological context was re-highlighted, and fine structures covered by the additive noise were restored as a result of noise removal. Further, compared with other classical methods (RSNLMMSE, PRI-NLM3D, BM4D, and HOSVD-R), our proposed algorithm can emphasize the lesion edge more effectively and preserve more small structures, which may assist the physician in decision making under complex conditions when images were acquired with heavy noise and play a key role for accuracy improvements in computer-aided detection/diagnostic tools such as tumor segmentation and recognition.

### Denoising on Clinical Three-Dimensional Magnetic Resonance Images

We also validated the denoising performance of the proposed algorithm on clinical MR datasets downloaded from the publicly available Open Access Series of Imaging Studies (OASIS) database (http://www.oasis-brains.org) (42). The clinical datasets (brain images, T1w) were of size 256 × 256 × 128 with a resolution of 1.0 *mm*^3^ × 1.0 *mm*^3^ × 1.25 *mm*^3^. The obtained MR images were originally corrupted by a certain level of clinical noise through acquisition. The Rician noise level was investigated according to (38), and the noise estimates for these MR images (OAS1_0092 and OAS1_0112) were ~4.5 and 3% of the maximum intensity, respectively.

**Figure 8:**
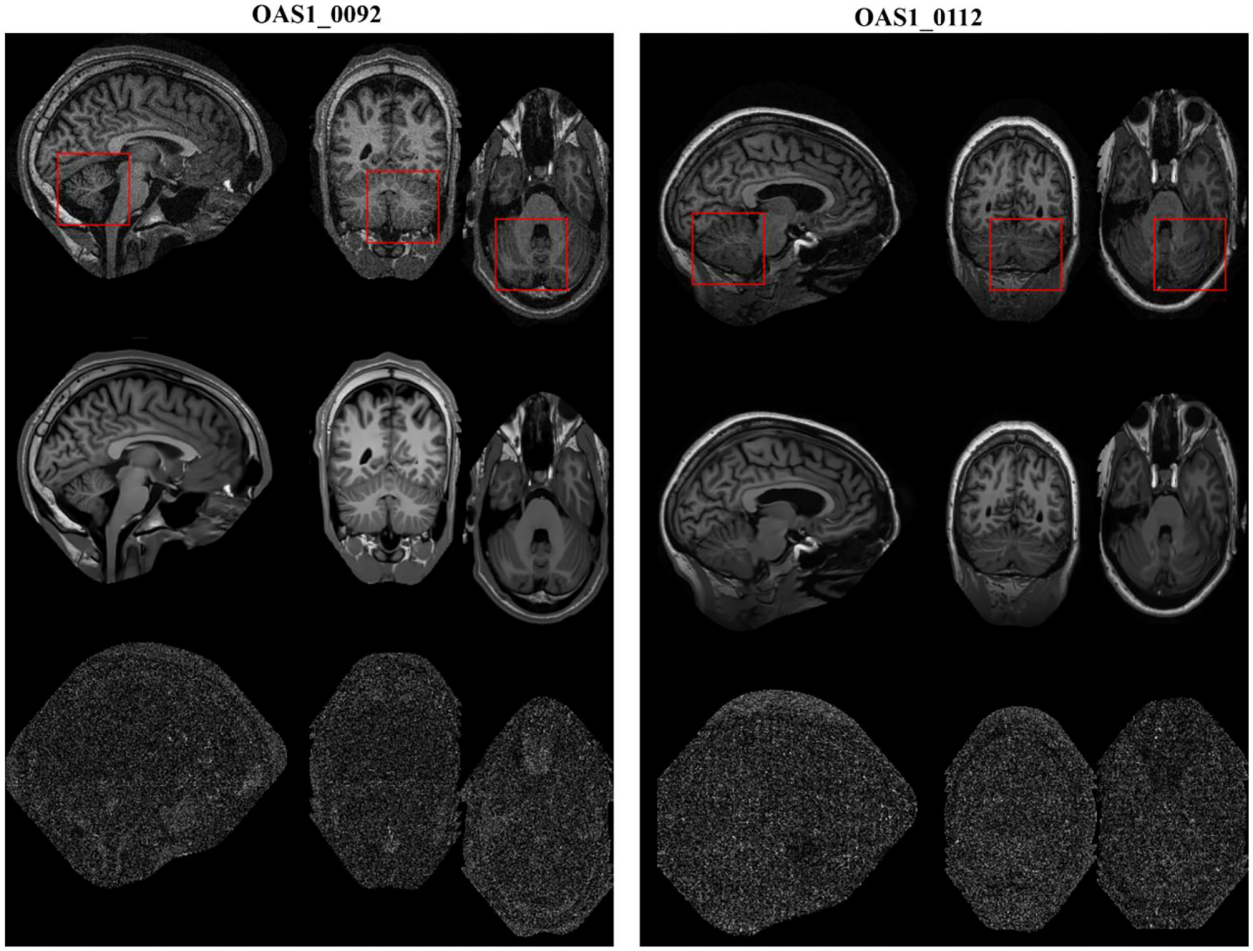
Example of clinical image denoising using the proposed method. First line: clinic noise image. Second line: the denoised images with the proposed method. Third line: the corresponding error images (the absolute difference between the denoised and noise images). Regions of interest (ROIs) (red rectangle) were selected and enlarged for better detail representation in this figure.

**Figure 9:**
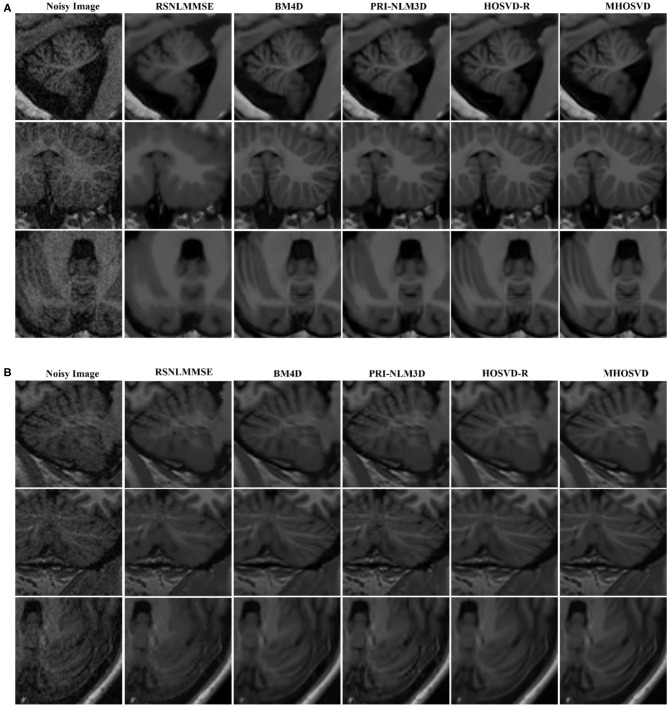
Denoising performances comparison based on regions of interest (ROIs) of top images (red rectangle in Figure 8). **(A)** Enlarged ROI from OAS1_0092. **(B)** Enlarged ROI from OAS1_0112. The proposed algorithm (*MHOSVD*) was compared with existing state-of-the-art methods including RSNLMMSE, BM4D, PRI-NLM3D, and HOSVD-R.

The restored results of our proposed *MHOSVD* for T1w brain images in sagittal, coronal, and transverse planes are presented in Figure 8. Further comparisons across four different algorithms (RSNLMMSE, BM4D, PRI-NLM3D, HOSVD-R, and *MHOSVD*) are shown in Figure 9. According to performance comparison (Figure 9), the statistical approach RSNLMMSE can restore the images but also severely erased structural details, and the PRI-NLM3D also showed some blurry edge and several tiny structures loss after noise removal. Again, based upon clinical images, the outstanding noise-reduction performance with excellent detailed structure reservation was practically observed using *MHOSVD*.

## Conclusions

In this study, we proposed an adaptive multilinear tensor rank approximation algorithm based on parameterized nonconvex logarithmic function for Rician noise removal in 3D MR images. The framework extracted 3D cube from noise images and searched similar cubes according to the Euclidean distance between cubes to construct the corresponding fourth-order tensor. The nonconvex logarithmic function was applied for the rank estimation of unfolding matrices along different modes of the tensor. Then the fourth-order tensors can be effectively recovered through the adaptive multilinear tensor rank approximation. Afterwards, the recovered cubes were aggregated to obtain the recovered volumetric images. Finally, we verified our algorithm on synthetic and clinical MR images. The proposed method exhibited a state-of-the-art performance in Rician noise removal with excellent fine detail preservation, which even outperformed several existing classical denoising methods (RSNLMMSE, BM4D, PRI-NLM3D, and HOSVD-R). Further, the capability to effectively capture the sparsity of multidimensional dataset would enable our work to be extended to several other domains, such as hyperspectral images (43, 44), color images (45), and video (46).

## Data Availability Statement

The image dataset testing the algorithm effectiveness and efficiency was originally accessed from Open Access Series of Imaging Studies (OASIS) database (http://www.oasis-brains.org).

## Author Contributions

LW designed the experiment, collected and analyzed the data, and drafted the manuscript. DX and XW participated in experiment setup and interpreted the results partly. LC and WH supervised the project and revised the manuscript. All listed authors approved the final manuscript.

## Conflict of Interest

The authors declare that the research was conducted in the absence of any commercial or financial relationships that could be construed as a potential conflict of interest. The handling editor declared a shared affiliation with the authors at the time of review.
